# Função Pulmonar e Força Muscular Inspiratória na Insuficiência Cardíaca: Elas Podem ser Consideradas Potenciais Marcadores Prognósticos?

**DOI:** 10.36660/abc.20211060

**Published:** 2022-04-07

**Authors:** Filipe Ferrari

**Affiliations:** 1 Faculdade de Medicina Universidade Federal do Rio Grande do Sul Hospital de Clínicas de Porto Alegre Porto Alegre RS Brasil Programa de Pós-Graduação em Cardiologia e Ciências Cardiovasculares, Faculdade de Medicina, Universidade Federal do Rio Grande do Sul, Hospital de Clínicas de Porto Alegre, Porto Alegre, RS – Brasil; 2 CardioEx Universidade Federal do Rio Grande do Sul Hospital de Clínicas de Porto Alegre Porto Alegre RS Brasil Grupo de Pesquisa em Cardiologia do Exercício (CardioEx) – Universidade Federal do Rio Grande do Sul, Hospital de Clínicas de Porto Alegre, Porto Alegre, RS – Brasil

**Keywords:** Insuficiência Cardíaca, Insuficiência Respiratória, Músculos Respiratórios, Função Ventricular, Tolerância ao Exercício, Medição de Risco

A insuficiência cardíaca (IC) é uma síndrome complexa considerada um grande problema de saúde pública. Diferentes subtipos de IC são classicamente definidos com base na fração de ejeção do ventrículo esquerdo (FEVE).^1^ Embora seu prognóstico tenha melhorado nas últimas décadas – explicado, em parte, pelos grandes avanços terapêuticos^2^ – a IC persiste com uma alta mortalidade influenciando negativamente a qualidade de vida.^3,4^ Nesse sentido, sintomas comuns experimentados nessa doença, como a falta de ar e a intolerância ao exercício, contribuem muito para esse declínio acentuado na qualidade de vida dos indivíduos.^5^ Outra condição, considerada um importante fator de risco que geralmente acompanha a IC, é a disfunção pulmonar.^6^ Os comprometimentos respiratórios observados na IC podem estar relacionados a diversas razões, como comprometimento da mecânica pulmonar e da difusão gasosa,^7^ além de fraqueza muscular respiratória – agravando o aumento da dispneia, sendo uma das principais limitações ao exercício físico.^8^

A espirometria é um teste amplamente utilizado que permite a análise da função pulmonar – medindo a quantidade de ar inspirado e expirado ao máximo. Como a doença pulmonar obstrutiva crônica compartilha sinais e sintomas semelhantes aos da IC, sua identificação em indivíduos com IC pode ser um desafio; nesse sentido, a espirometria pode ajudar a confirmar o diagnóstico.^9^ Na avaliação da gravidade potencial de algumas doenças pulmonares, o teste ergométrico também pode ser útil, observando uma série de parâmetros, como a relação volume expiratório forçado no primeiro segundo/capacidade vital forçada (VEF_1_/CVF).^10^ A gravidade da doença ainda pode ser classificada com base no VEF_1_ quando está abaixo do limite inferior da normalidade (variando de leve quando ≥70% do previsto a muito grave quando <35% do previsto). Embora a própria IC possa levar a uma diminuição do VEF_1_ e da CVF em cerca de 20% do previsto,^10^ além do fato de que um VEF_1_ pior pode predizer maior mortalidade,^11^ evidências convincentes que examinem o papel prognóstico do VEF_1_ no cenário da IC ainda precisam de mais investigação.

Nesta edição dos Arquivos Brasileiros de Cardiologia, Ramalho et al.^12^ compartilharam dados de um estudo de coorte com 111 adultos brasileiros (média de idade: 57 anos; 40% mulheres) com IC crônica, sem doença pulmonar diagnosticada e que realizaram teste de força muscular respiratória e espirometria; os participantes foram posteriormente acompanhados por uma média de 2,2 anos. Alguns dos objetivos do estudo foram analisar a relação VEF_1_/CVF com (a) pressão inspiratória máxima, (b) FEVE e (c) prognóstico dos pacientes – este último definido como um composto de morte cardiovascular (CV), transplante cardíaco de emergência ou implante de dispositivo de assistência ventricular esquerda. No geral, a FEVE média inicial foi de 38%, mas 24 dos pacientes apresentaram FEVE >50%; a grande maioria da amostra (64%) estava na classe III pela classificação da NYHA. A cardiopatia isquêmica e a doença de Chagas foram as principais etiologias observadas (39% e 29%, respectivamente). Os pacientes estavam relativamente bem tratados, recebendo terapia médica otimizada (betabloqueadores em 90%, inibidores do sistema renina-angiotensina-aldosterona em 84% e antagonistas dos receptores mineralocorticoides em 66%).

Este artigo tem várias descobertas interessantes que merecem destaque. Tanto a CVF quanto a VEF_1_/CVF não se correlacionaram com melhor ou pior prognóstico durante o seguimento médio. Por outro lado, após uma análise de sensibilidade, uma VEF_1_/CVF baixa foi indicada como um potencial marcador de risco para aumento de eventos adversos cardiovasculares maiores nos indivíduos teoricamente mais graves, ou seja, com FEVE <50%. Além disso, um risco maior de eventos cardiovasculares foi observado naqueles com pressão inspiratória máxima reduzida e VEF_1_/CVF (razão de risco 1,72; intervalo de confiança de 95%, 1,14 a 2,61).

Décadas atrás, Tockman et al.^13^ relataram o VEF_1_ como um preditor independente de mortalidade CV após acompanhar uma coorte de homens aparentemente saudáveis. Em outros estudos observacionais que avaliaram o prognóstico da pressão inspiratória máxima em pacientes com IC, Hamazaki et al.^14^ relataram menor incidência de eventos clínicos em pacientes com grande variedade de FEVE (maioria na classe funcional II da NYHA) quando uma pressão inspiratória máxima maior estava presente, após sessões de reabilitação cardíaca e com seguimento médio de 1,8 anos, mesmo após ajuste para fatores de confusão. Meyer et al. sugeriram que a força muscular inspiratória poderia ser útil na estratificação de risco dos pacientes.^15^

Apesar dos achados interessantes, que em certa medida corroboram estudos anteriores, o estudo de Ramalho et al.^12^ não permite inferências causais com segurança devido ao seu desenho observacional e deve ser interpretado à luz desta e de outras possíveis limitações. Embora esteja bem estabelecido que a IC é comumente caracterizada por anormalidade dos músculos respiratórios, com consequente declínio na qualidade de vida e possivelmente pior prognóstico, seria prematuro concluir definitivamente uma associação direta entre a pressão inspiratória máxima ou VEF_1_/CVF com risco aumentado de eventos cardiovasculares nesta população, independentemente da FEVE. Apesar destes comentários, este estudo fornece informações importantes para a literatura e reacende a possibilidade de que a VEF_1_/CVF possa ser usada como ferramenta prognóstica, oferecendo informações incrementais no cenário da IC, especialmente no grupo de pacientes considerados de maior risco. Ainda assim, seria prudente afirmar que a relação entre esses marcadores e o prognóstico desses indivíduos permanece incerta.
