# Supplementary material for: Função Pulmonar e Força Muscular Inspiratória na Insuficiência Cardíaca: Elas Podem ser Consideradas Potenciais Marcadores Prognósticos?
Source: Arq Bras Cardiol. 2022 Apr 7;118(4):692–3. [Article in Portuguese] doi: 10.36660/abc.20211060 (PMC9007001; doi:10.36660/abc.20211060)
Supplement: Translation

# Translation: Lung Function and Inspiratory Muscle Strength in Heart Failure: Can They be Considered Potential Prognostic Markers?

**Authors:** Filipe Ferrari

**Affiliations:** 1 Brazil Programa de Pós-Graduação em Cardiologia e Ciências Cardiovasculares, Faculdade de Medicina, Universidade Federal do Rio Grande do Sul, Hospital de Clínicas de Porto Alegre, Porto Alegre, RS – Brazil; 2 Brazil Grupo de Pesquisa em Cardiologia do Exercício (CardioEx) – Universidade Federal do Rio Grande do Sul, Hospital de Clínicas de Porto Alegre, Porto Alegre, RS – Brazil

**Keywords:** Heart Failure, Respiratory Insufficiency, Respiratory Muscles, Ventricular Function, Exercise Tolerance, Risk Assessment

Heart failure (HF) is a complex syndrome considered a major public health problem. Different HF subtypes are classically defined based on left ventricular ejection fraction (LVEF).^1^ Although its prognosis has improved in recent decades – explained, in part, by major therapeutic advances^2^ – HF persists with a high burden of mortality and negatively influences the quality of life.^3,4^ In this sense, common symptoms experienced in this disease, such as shortness of breath and exercise intolerance, contribute greatly to this sharp decline in the quality of life of individuals.^5^ Another condition, considered an important risk factor, which usually accompanies HF, is pulmonary dysfunction.^6^ The respiratory impairments observed in HF may be related to several reasons, such as an impairment of lung mechanics and gas diffusion,^7^ as well as respiratory muscle weakness – aggravating increased dyspnea, being a major limitation on physical exercise.^8^

Spirometry is a widely used test that allows the analysis of lung function – by measuring the amount of air inhaled and exhaled to the maximum. Because chronic obstructive pulmonary disease shares similar signs and symptoms with HF, its identification in individuals with HF can be challenging; in this sense, spirometry can help confirm the diagnosis.^9^ About evaluating the potential severity of some lung diseases, the exercise test can also be useful, observing a series of parameters, such as the ratio of forced expiratory volume in 1 second/forced vital capacity (FEV_1_/FVC).^10^ The severity of the disease can still be classified based on FEV_1_ when it is below the lower limit of normal (ranging from mild when ≥70% of predicted to very severe when <35% of predicted). Although HF itself can lead to a decrease in FEV_1_ and FVC of about 20% of predicted,^10^ in addition to the fact that a worse FEV_1_ may predict higher mortality,^11^ convincing evidence examining the prognostic role of FEV_1_ in the HF setting still needs further investigation.

In this issue of the ABC, Ramalho et al.^12^ shared data from a cohort study of 111 Brazilian adults (mean age: 57 years; 40% women) with chronic HF, no diagnosed lung disease, and who underwent a test of respiratory muscle strength and spirometry; participants were subsequently followed for a mean of 2.2 years. Some of the aims of the study were to analyze the FEV1/FVC ratio with (a) maximal inspiratory pressure, (b) LVEF, and (c) patients’ prognosis – the latter being defined as a composite of cardiovascular (CV) death, emergency heart transplantation or left ventricular assist device implantation. Overall, the initial mean LVEF was 38%, but 24 of the patients had an LVEF >50%; the vast majority of the sample (64%) was in class III using the NYHA classification. Ischemic heart disease and Chagas’ disease were the main etiologies observed (39% and 29%, respectively). The patients were relatively well treated, receiving optimal medical therapy (beta-blockers in 90%, renin-angiotensin-aldosterone system inhibitors in 84%, and mineralocorticoid receptor antagonists in 66%).

This article has several interesting findings worth highlighting. Both FVC and FEV_1_/FVC did not correlate with a better or worse prognosis during the mean follow-up. On the other hand, after a sensitivity analysis, a low FEV_1_/FVC was indicated to be a potential risk marker for increased major adverse CV events in the theoretically more severe individuals, i.e., with LVEF <50%. Furthermore, a higher risk of CV events was observed in those with both reduced maximal inspiratory pressure and FEV_1_/FVC (hazard ratio 1.72; 95% confidence interval, 1.14 to 2.61).

Decades ago, Tockman et al.^13^ reported FEV_1_ as an independent predictor for CV mortality after following a cohort of apparently healthy men. In other observational studies that evaluated the prognosis of maximal inspiratory pressure in patients with HF, Hamazaki et al.^14^ reported a lower incidence of clinical events in patients with a wide variety of LVEF (majority in NYHA functional class II) when a higher maximal inspiratory pressure was present, after cardiac rehabilitation sessions and at a mean follow-up of 1.8 years, even after adjustment for confounding factors. Meyer et al. suggested that inspiratory muscle strength could be useful in risk stratification of patients.^15^

Despite interesting findings, which to some extent corroborate previous studies, the study by Ramalho et al.^12^ does not allow us to make causal inferences safely due to its observational design. It should be interpreted in light of this and other possible limitations. Although it is well established that HF is commonly characterized by abnormality of respiratory muscles, the consequent decline in quality of life, and possibly worse prognosis, it would be premature to definitively conclude a direct association between maximal inspiratory pressure or FEV_1_/FVC with increased risk of CV events in this population, regardless of LVEF. Despite these comments, this study provides important information to the literature, and it rekindles the possibility that FEV_1_/FVC can be used as a prognostic tool, offering incremental information in the HF scenario, especially in the group of patients considered at higher risk. Still, it would be prudent to state that the relationship between these markers and the prognosis of these individuals remains uncertain.
